# Isolation, purification and characterization of 5'-phosphodiesterase from *Aspergillus fumigatus*

**DOI:** 10.1371/journal.pone.0186011

**Published:** 2017-10-26

**Authors:** Zhiting Luo, Yingying Fan, Qiuxia Li, Bing Han, Yang Liu, Shubo Li, Hua Qiu, Zongwen Pang

**Affiliations:** 1 College of Light Industry and Food Engineering, Guangxi University, Nanning, China; 2 College of Life Science and Technology, Guangxi University, Nanning, China; 3 Liquor Making Biological Technology and Application of key laboratory of Sichuan Province, Zigong, China; Russian Academy of Medical Sciences, RUSSIAN FEDERATION

## Abstract

5′-Phosphodiesterase (5′-PDE) catalyzes the hydrolysis of ribonucleic acid to obtain a mixture of ribonucleotides, such as 5′-guanosine monophosphate and 5′-adenosine monophosphate. In this study, a 5'-PDE was newly isolated and purified from *Aspergillus fumigatus*. Following purification, this enzyme exhibited a specific activity of 1036.76 U/mg protein, a molecular weight of 9.5 kDa, and an optimal temperature and pH for enzyme activity of 60°C and 5.0, respectively. However, its activity was partially inhibited by Fe^3+^, Cu^2+^, and Zn^2+^, but slightly improved by the presence of K^+^ and Na^+^. Additionally, chemical-modification experiments were also applied to investigate the structural information of 5'-PDE, in which the residues containing carboxyl and imidazole groups were essential for enzyme activity based on their localization in the 5′-PDE active site. Furthermore, purified 5′-PDE could specifically catalyze the synthesis of ribonucleotides with a *V*_max_ 0.71 mmol/mg·min and a *K*_M_ of 13.60 mg/mL.

## Introduction

Recently, interest in 5′ nucleotides has increased due to their applications in food and the pharmaceutical industries [1]. As a flavor enhancer, 5′ nucleotides are capable of effectively improving the taste of food and exhibit synergistic effects with monosodium glutamate [2]. Additionally, 5′ nucleotides can be also used to synthesize antivirus and anticancer drugs as an intermediate, with its derivatives also having important uses for the treatment of illness of the human central nervous and circulatory systems[3]. There are currently three methods for producing nucleotides, including microbial fermentation, chemical synthesis, and enzymatic methods [4]. Compared to other methods, use of enzymes has incomparable advantages, such as low cost, low complexity, and high yield, making their use the most economical method in the industry [5]. Therefore, it is important to obtain an excellent enzyme capable of producing 5′ nucleotides.

As a commercial 5′-nucleotide-producing enzyme, 5′-phosphodiesterase (5′-PDE; EC 3.1.4.1) can cleave RNA into 5′-mononucleotides (e.g., 5′-AMP, -GMP, -CMP, and -UMP) that are frequently used as additives in the food industry [6, 7], auxiliary therapy for hepatitis, nephritis, and muscle diseases [8], and agents in molecular biology research [9]. Currently, 5′-PDE is purified and obtained from several sources, including microbes, such as *Penicillium citrinum* [10] and *Streptomyces aureus* [11], plant sources, such as *Catharanthus roseus* [12], mung bean, barley rootlets, and seeds [13], and animal sources, such as snake venom phosphodiesterase and bovine intestine kidney [14]. Given the economic constraints associated with processing and the ease of large-scale production, microbial sources are generally preferred as the sources of these enzymes.

Currently, malt-root extraction and *P*. *citrinum*-based fermentation are used as the main 5′-PDE production methods, but are also constrained due to the high content of impurities and the low activity of 5′-PDE[2, 15]. Therefore, high-producing strains for 5′-PDE are imperative to enhance nucleotide production. Here, a strain demonstrating high productivity of 5′-PDE from *Aspergillus fumigatus* was isolated and verified according to its 18S rDNA sequence. Following purification, its enzyme characteristics and structural information were also investigated, demonstrating that the excellent properties for RNA hydrolysis.

## Materials and methods

### Ethics statement

This study was conducted at the soil and agriculture residues in Nanning, Guangxi Guangxi Province, China. These locations are located at approximately 108^o^22' E and 22^o^48'N, and not nationally protected and therefore no specific permissions were required. Field work was non-extractive (collection and observations data) and did not involve removal of endangered or protected species on any site.

### Materials

RNA (from yeast), AMP, GMP, CMP, UMP, and IMP were purchased from Sigma–Aldrich (Steinheim, Germany). DNA polymerase, ligase, and a genomic DNA purification kit were purchased from Sangon (Shanghai, China). All other compounds were of reagent grade or higher quality.

### Media and culture conditions

Medium A was used to enrich the 5′-PDE-producing strain and contained 5 g/L RNA, 0.1 g/L MgSO_4_, 1 g/L K_2_HPO_4_, 0.01 g/L FeSO_4_, 0.05 g/L KCl, and 12 g/L agar. Potato Dextrose Agar media (PDA, Medium B) was used for seed culture. Medium C was used for 5′-PDE fermentation and contained 1000 g/L bran, 0.3 g/L zinc sulfate, 0.4 g/L calcium carbonate, and 1 g/L potassium dihydrogen phosphate.

For 5′-PDE production, bacteria were grown at 30°C in medium B for 7 days, followed transfer of 1 mL spore suspension (0.2% NaCl) to inoculate the fermentation medium in Erlenmeyer flasks (50 mL/500 mL) at 30°C with 200 rpm.

### Isolation and identification of the AMP deaminase-producing strain

200 samples were collected from soil and agriculture residues in Nanning, Guangxi Guangxi Province, China. Samples (2 g) were added to tubes containing 20 mL of medium A and mixed thoroughly. After incubating at 30°C for 72 h, GMP, AMP, and 5′-PDE contents were measured. Samples containing 5′-PDE were diluted 100-, 1000, and 10,000-fold and spread onto screening plates containing medium B for incubation at 30°C for 72 h. Colonies that appeared were selected and transferred to medium C for inoculation at 30°C for 72 h to determine 5′-PDE-production capability. Strains that produced high levels of 5′-PDE were preserved in sand tubes for long-term storage. These 5′-PDE-producing strains were identified by physiological characteristics according to 18S rDNA sequence analysis.

### 5′-PDE purification

After a 72-h incubation at 30°C under solid-state fermentation, the starter culture was harvested and dipped in 10× water for 8 h, and then centrifugated at 12,000 *g* for 10 min at 4°C to obtain the supernatant. To purify the enzyme, the supernatant was first isolated by microfiltration (Sangon Biotech, China) with a 0.22 μm membrane, ultrafiltration with 10- and 4-kDa membranes, and then precipitated by ammonium sulfate and separated by diethylaminoethyl (DEAE)-cellulose ion-exchange chromatography and DEAE-sepharose CL-6B ion-exchange chromatography (Sigma-Aldrich, Germany). Protein purity was determined by sodium dodecyl sulfate polyacrylamide gel electrophoresis (SDS-PAGE) analysis. Protein concentrations were measured by Bradford method [16], and concentrations during purification studies were calculated from the standard curve.

### Enzyme assays

5′-PDE activity was measured by a spectrophotometric method as previously described [17, 18]. A certain concentration (T) of enzyme solution (0.1 mL) was incubated with substrate solution [1% RNA (w/v) and 0.125 M acetate buffer (pH 5.0)] at 60°C for 10 min and stopped by adding 2.0 mL of ice-cold nucleic acid precipitator (0.25% ammonium molybdate dissolved in 2.5% perchloric acid) for 25 min in an ice bath. After removing the precipitated RNA by centrifugation at 5000 g for 5 min at 4°C, 1 ml of the supernatant was diluted into 50 mL with distilled water, and its absorbance was measured with a UV-vis absorbance detector (Hewlett-Packard, Waldbrom, Germany) at 260 nm using a blank incubation in the absence of enzyme as the control. One unit of enzyme activity was defined as the amount of enzyme that produced an increase in optical density of 1 after 1 min at 260 nm, and the activity (U) of 5′-PDE was calculated as follows:

$$Enzyme\;activity\;\left(U/mL\right)=(△A_{260}\times 4\times 50\times T)/\left(0.1*15\right)=△A_{260}\times T\times 133.3$$ (1)

where △A_260_ is the difference in absorbance between enzymatic reaction and the control, T is the dilution factor of the enzyme prior to the start of the assay, 4 is the volume of reaction system, 50 is the dilution ratio after finishing reaction, 0.1 is the volume of enzyme solution, 15 is the reaction time.

### Enzyme kinetics

The optimal pH was determined in the pH range of 3.5 to 7.0 using different buffers, including acetate buffer (pH 3.5–6.0) and phosphate buffer (pH 6.0–7.0). Under the optimal pH 5.0, the effect of temperature on 5′-PDE activity was determined at temperatures ranging from 40°C to 70°C with 5°C increases. 5′-PDE activity of at each point was normalized as the percent of highest enzyme activity at the corresponding pH and temperature.

To determine the effect of different metal ions on 5′-PDE, the enzyme was treated with various metal ions (Mg^2+^, Mn^2+^, Ca^2+^, Zn^2+^, Ni^2+^, Fe^3+^, or Al^3+^; 1 mmol/L) at 30°C for 30 min, followed by measurement of residual activity.

Apparent 5′-PDE kinetic parameters were measured using reaction mixtures containing variable amounts of RNA (1.0–12.0 mg/mL) in 0.1 mol/L acetate buffer (pH 5.0). *K*_M_ and *V*_max_ values were calculated using Lineweaver–Burk plotting plots.

### Modification of 5′-PDE activity by different chemicals

#### Modification by carbodiimide (EDC)

Different concentrations of EDC (10, 50, 250, 500, and 1000 mmol/L) were dissolved in 50 mM acetate buffer (pH 5.0), and the enzyme solution (0.1 mL) was incubated with substrate solution (0.1 mL EDC in 0.8 mL acetate buffer) at 30°C for 30 min. The remaining 5'-PDE activity of at each point was normalized as the percentage of enzyme activity in the absence of EDC. Similarly, the reaction system [0.1 mL enzyme solution, 0.1 mL EDC, 0.2 mL acetate buffer, and 0.6 mL of 1% RNA (w/v)] was used to detect the effect of substrate on EDC regarding 5′-PDE activity.

#### Modification by 2,4,6-trinitrobenzenesulfonic acid sol (TNBS)

Different concentrations of TNBS (0.1, 1, 5, 10, and 20 mmol/L) were dissolved in 100 mM phosphate buffer (pH 8.0), and the enzyme solution (0.1 mL) was incubated with substrate solution (0.1 mL TNBS and 0.8 mL phosphate buffer) at 30°C for 30 min. The remaining activity of 5′-PDE at each point was normalized as the percentage of enzyme activity in the absence of TNBS. Similarly, the reaction system [0.1 mL enzyme solution, 0.1 mL TNBS, 0.2 mL phosphate buffer, and 0.6 mL of 1% RNA (w/v)] was applied to detect the effect of substrate on TNBS regarding 5'-PDE.

Similarly, different concentrations of diethyl oxydiformate [DEPC; using 100 mM phosphate buffer (pH 7.4)], 2,3-butanedione [DIC; using 50 mM borate buffer (pH 8.5)], *N*-ethylmaleimide [NEM; using 100 mM phosphate buffer (pH 7.4)], phenylmethanesulfonyl fluoride [PMSF; using 100 mM phosphate buffer (pH 7.4)], and 2-hydroxy-1-ethanethiol [2-ME; using 100 mM phosphate buffer (pH 8.0)] were also applied to modify 5′-PDE activity to investigate the structural characteristics of 5′-PDE.

### Analysis of 5′-PDE reaction products

The total concentrations of AMP, GMP, and UMP in the reaction mixture were determined by high-performance liquid chromatography (HPLC; Dionex UltiMate 3000 Series, Thermo Fisher Scientific, Waltham, MA, USA) equipped with a Synergi Fusion-RP C18 column (250 × 4.60 mm) and an ultraviolet detector (λ = 260 nm), with 0.1 mol/L KH_2_PO_4_ used as the mobile phase at a flow rate of 0.6 mL/min. All experiments were performed in triplicate, and data are represented as the average of three replicates, with errors indicating the standard error of the mean.

## Results

### Isolation and identification of the 5′-PDE-producing strain

In this study, RNA was used as a sole carbon and nitrogen source for isolating 5′-PDE-producing strains, in which 100 samples were diluted and transferred to plates with medium A for incubation under aerobic conditions. Colonies that grew on medium A plates were inoculated into medium B to assess 5′-PDE-producing capabilities. As shown in Table 1, 12 strains were able to synthesize and secrete >500 U/g 5′-PDE, with strain XD-9 secreting the highest quantity (2014.5 U/g 5′-PDE) and selected for subsequent 5′-PDE-specific fermentation. According to the 18S rDNA sequence, strain XD-9 shared 99% sequence identity with strains of *A*. *fumigatus* SCSGAF0014 (JN850983) and *A*. *fumigatus* (HQ149773) (S1 Fig). Phylogenetic experiments demonstrated that strain XD-9 belonged to *A*. *fumigatus*, which was subsequently named *A*. *fumigatus* XD-9.

**Table 1:** The production of 5'-PDE from isolates in solid-state fermentation.

Strains	5'-PDE (U/g dried culture)	Strains	5'-PDE (U/g dried culture)
**XD-1**	502.7 ± 20.1	YL-1	521.3 ± 32.1
**XD-2**	536.2 ± 10.8	YL-2	532.3 ± 14.5
**XD-3**	595.5 ± 15.0	YL-3	685.3 ± 9.9
**XD-4**	524.2 ± 27.1	YL-4	714.6 ± 24.9
**XD-5**	527.2 ± 8.5	YJ-1	531.3 ± 17.3
**XD-6**	636.3 ± 22.4	YJ-2	607.3 ± 28.1
**XD-7**	798.8 ± 30.4	YJ-3	965.4 ± 25.9
**XD-8**	993.9 ± 33.5	YJ-4	586.7 ± 30.5
**XD-9**	2014.5 ± 22.8	YJ-5	1052.1 ± 45.0
**XD-10**	1162.4 ± 12.3	MS-1	530.7 ±12.4
**XD-11**	1110.3 ± 25.5	MS-2	526.7 ± 18.0
**XD-12**	1329.3 ± 11.5	MS-3	1140.5 ± 17.5

### Optimization of 5′-PDE production through orthogonal experiments

To optimize 5′-PDE production, different factors, including carbon source, nitrogen source and temperature, were investigated during preliminary experiments, where glucose and water content affected 5′-PDE production. Therefore, different levels of glucose and water content were employed, resulting in maximum 5′-PDE titers from 40 g/L glucose and 45% water content of 3753.4 U/g and 4512.6 U/g, respectivity (S2 Fig). Subsequently, the optimal levels of glucose and water content were investigated with an orthogonal array described in Table 2, resulting in production of 5783.9 U/g 5′-PDE from 40 g/L glucose and 44% water content (experimental combination 5).

**Table 2:** Orthogonal array design for 5'-PDE production.

Run	Factors		A Glucose (g/L)	B Water content (%)	5'-PDE (U/g dried culture)
	A	B			
1	1	1	35	42	3277.0
2	1	2	35	44	3722.8
3	1	3	35	46	3098.5
4	2	1	40	42	4371.6
5	2	2	40	44	5783.9
6	2	3	40	46	3994.8
7	3	1	45	42	3753.8
8	3	2	45	44	3552.2
9	3	3	45	46	3360.8
*K*_1_			3366.1	3800.8	
*K*_2_			4716.8	4352.9	
*K*_3_			3555.6	3484.7	
*R*			1350.667	868.267	
*Q*			*A*_2_	*B*_2_	

### 5′-PDE purification

To obtain the higher degree of 5′-PDE purity, 5′-PDE purification was performed by microfiltration, ultrafiltration, ammonium sulfate precipitation, DEAE-cellulose column chromatography, and DEAE-sepharose CL-6B chromatography (Table 3). Following purification, the total activity and amount of the crude 5′-PDE extract decreased significantly from 180,262.2 U to 14,100.4 U and 14,272.0 mg to 13.6 mg, respectively. Correspondingly, the specific activity of 5′-PDE increased markedly by ~82.1-fold, reaching 1036.8 U/mg protein with a yield of 7.8%. Additionally, purification results were also evaluated by nonreducing SDS-PAGE, which showed a single protein band at 9.5 kDa, indicating significant improvement in enzyme purity (S3 Fig).

**Table 3:** Summary of different methods for 5'-PDE purification.

Purification steps	Total activity (U)	Total protein (mg)	Specific activity (U/mg protein)	Purification (Fold)	Yield (%)
Supernatant	180,262.2	14,272.0	12.6	1.0	100.0
Microfiltration (0.22μL)	178,140.5	11,643.7	15.3	1.2	98.8
Ultrafiltration (10 K)	162,300.6	4507.6	36.1	2.9	90.0
Concentration (4K)	134,100.3	1601.5	83.8	6.6	74.4
Salting-out	95,720.2	456.1	209.9	16.6	53.1
DEAE-cellulose	36,300.7	54.4	672.2	53.2	20.1
DEAE-sepharoseCL-6B	14,100.4	13.6	1036.8	82.1	7.8

### Enzymatic properties

**Fig 1:**
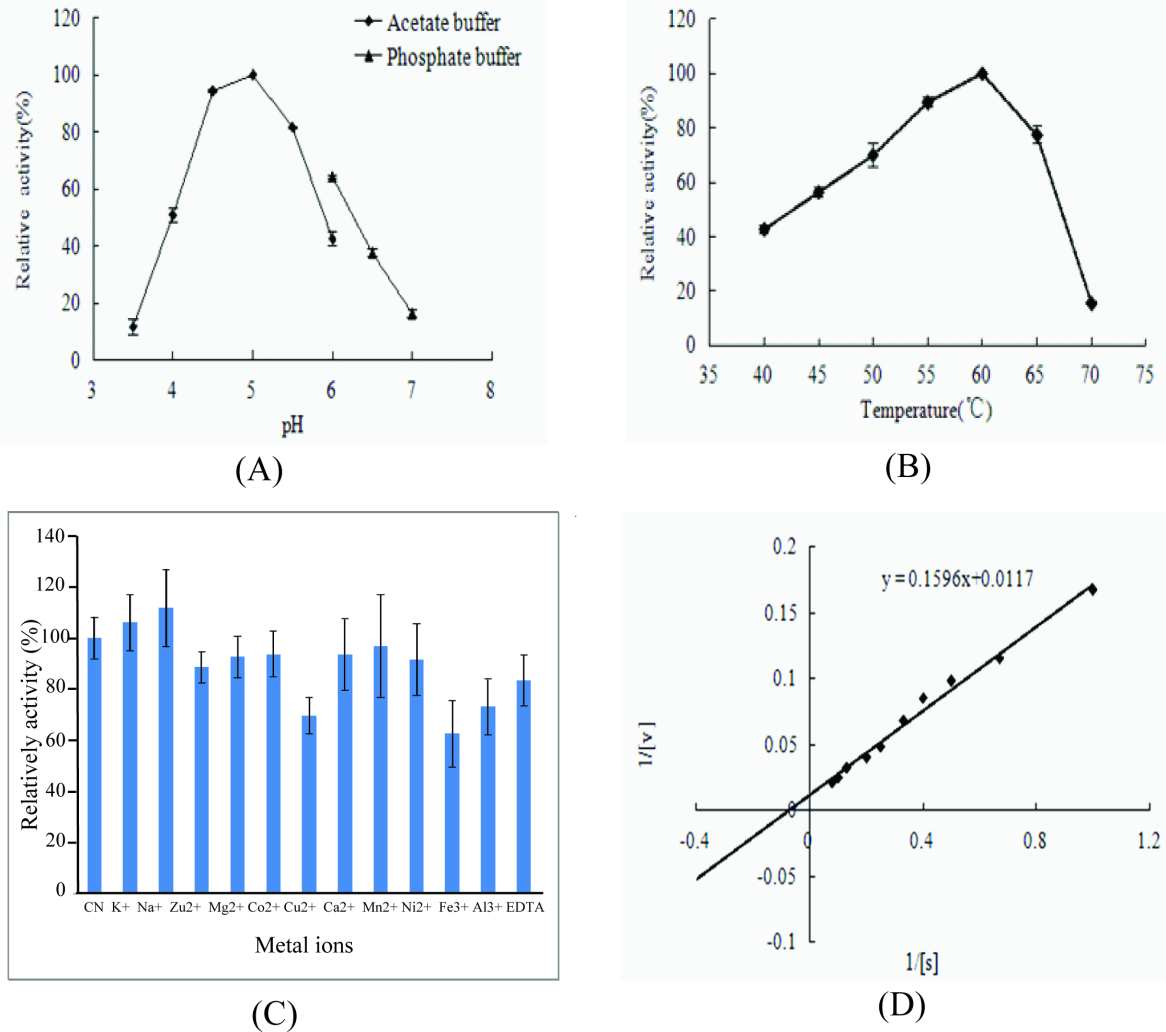
5′-PDE enzymatic properties. Effect of (A) pH (the relative activities at various pH levels were measured with several different buffer systems at 60°C), (B) temperature (the relative activities at different temperature were determined at pH 5.0), and (C) metal ions on 5′-PDE activity (determing at 60°C and pH 5.0). (D) *V*_max_ and *K*_M_ values according to Lineweaver–Burk plots. Each value represents the mean of three independent measurements.

As shown in Fig 1A, the relative activities of 5′-PDE at various pH levels were measured with several different buffer systems at 60°C, with the purified 5′-PDE showing the highest catalytic activity at pH 5.0. However, its activity was dramatically decreased at pH <4.5 or >5.5(Fig 1A). Furthermore, the effect of temperature on 5′-PDE activity was also determined at pH 5.0 (Fig 1B), revealing that enzyme activity gradually increased at temperatures between 40°C and 60°C, but sharply decreased at higher temperatures, indicating that the optimal temperature for 5′-PDE activity was 60°C.

Additionally, the effects of different metal ions on enzyme activity were also investigated (Fig 1C). Our results indicated that the presence of Fe^2+^, Fe^3+^, and Cu^2+^ inhibited 5′-PDE activity, whereas K^+^ and Na^+^ slightly improved 5′-PDE activity. We observed that 0.1 mol/L Fe^2+^ or Cu^2+^ significantly decreased 5′-PDE activity by 34.5% and 26.2%, respectively. Under optimal conditions (60°C, pH 5.0, and 0.1 mol/L Na^+^), 5′-PDE kinetic parameters were measured using from 1.0 mg/mL to 12.0 mg/mL of RNA as substrate, resulting in apparent *K*_M_ and *V*_max_ values of 13.60 mg/mL and 0.71 mmol/mg·min, respectively (Fig 1D).

### 5′-PDE structural properties

To obtain structural information concerning the amino acid composition of 5′-PDE, chemicals, including EDC, DIC, NEM, and TNBS, were used to modify the 5′-PDE active site [19, 20].

#### Effects of modification with EDC or DEPC on 5′-PDE structural properties

**Fig 2:**
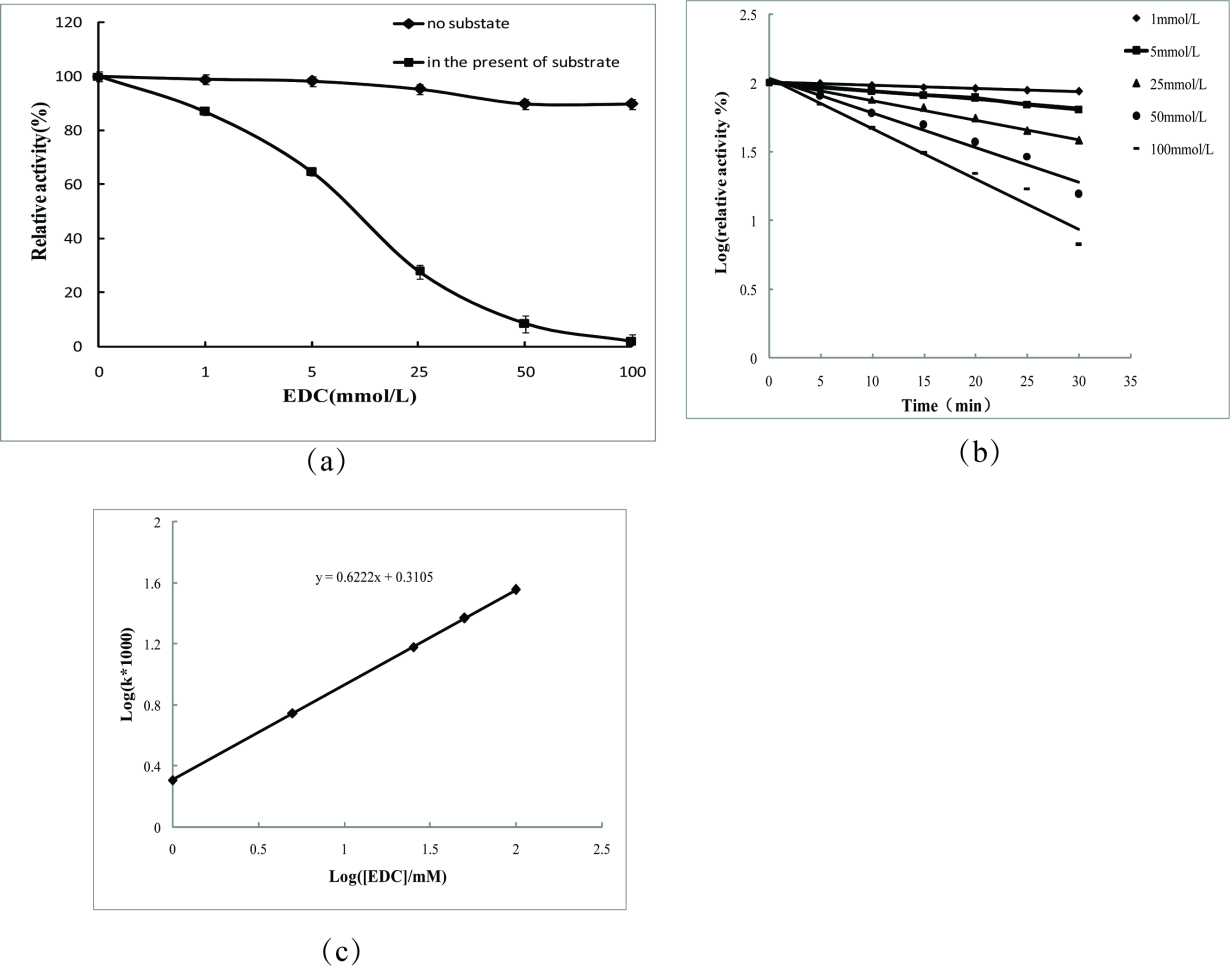
Effects of EDC modification on 5′-PDE activity. (A–C) Effect of EDC modification on 5′-PDE activity and kinetics.

Under acidic conditions, EDC was applied to modify carboxyl groups, such as those of glutamic acid and aspartic acid side chains. When in the absence of RNA, increasing EDC concentrations could significantly decrease 5′-PDE activity (Fig 2A). Upon increases of EDC concentration to 100 mmol/L, the relative activity of 5′-PDE was only 1.9%, suggesting that a residue containing a carboxyl group was located in the 5′-PDE active site. However, in the presence of RNA, the activity of 5'-PDE was only slightly decreased (90%) along with increased EDC concentration. These findings indicated that residues containing carboxyl groups were essential to maintaining 5′-PDE activity.

Additionally, to confirm the number of carboxyl group, the ability of EDC to alter 5′-PDE enzyme kinetics was also investigated. Our results showed that the relationship between the logarithm of relative enzyme activity and time (y = 0.6222x + 0.3105) suggested that one carboxyl group located in the 5′-PDE active site (Fig 2B and 2C).

**Fig 3:**
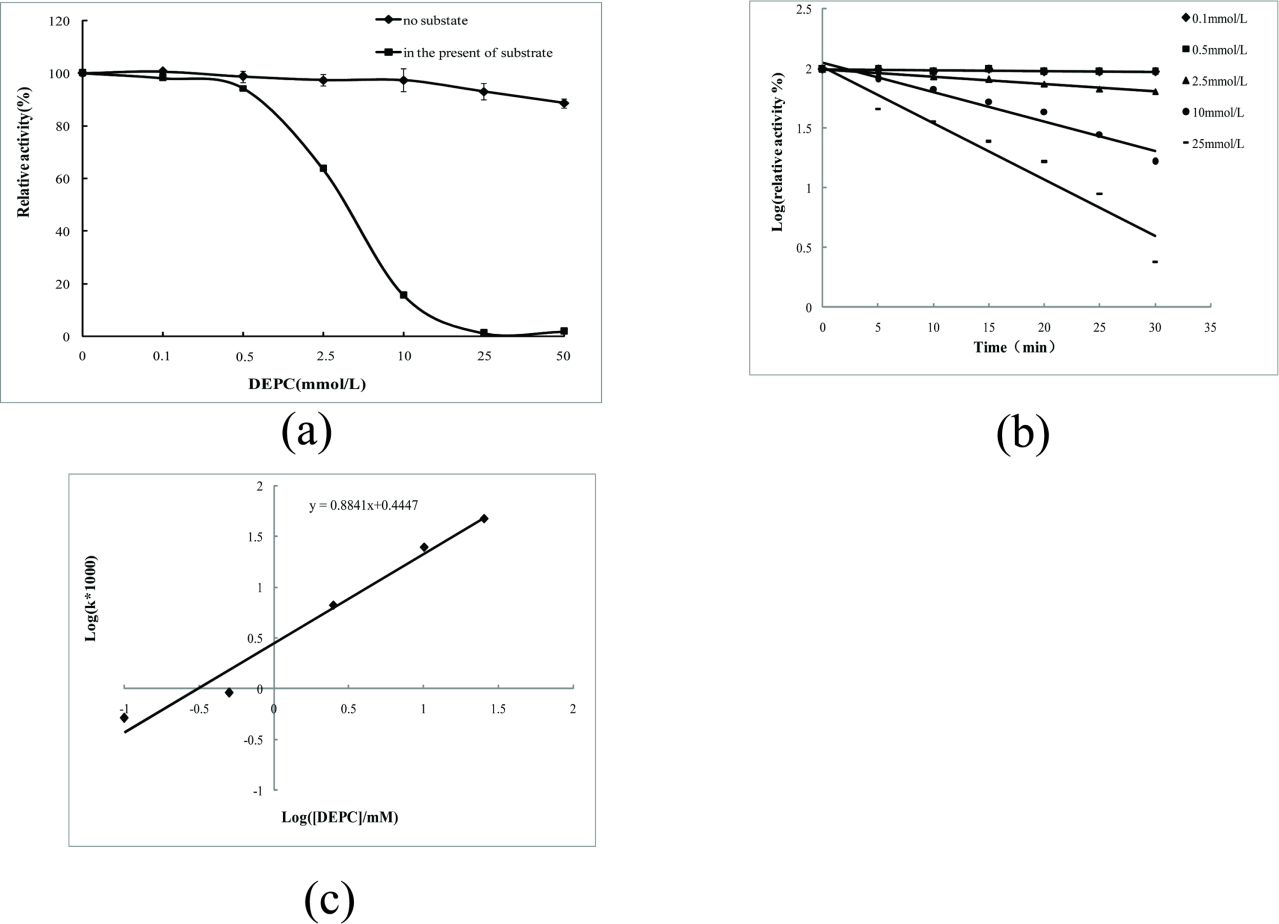
Effects of DEPC modification on 5′-PDE activity. (A–C) Effect of DEPC modification on 5′-PDE activity and kinetics.

Similarly, DEPC was also used to modify imidazole groups present in histidine residues under neutral conditions. Our findings demonstrated that one imidazole group was present the active site and essential for 5′-PDE activity (Fig 3).

#### Effects of modification with TNBS, DIC, or 2-ME on 5′-PDE structural properties

**Fig 4:**
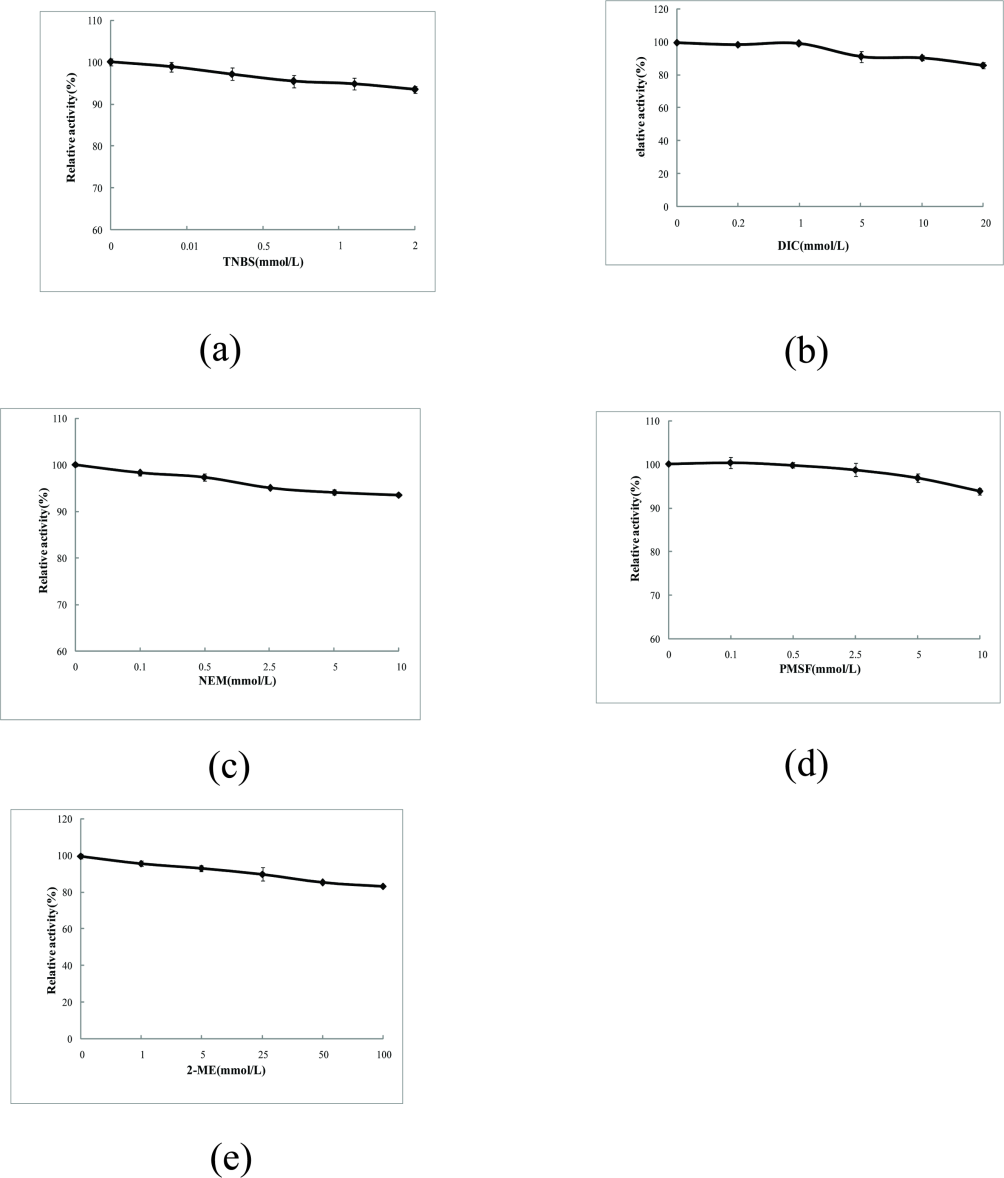
Effects of modification with different chemicals on 5′-PDE activity. Effects of (A) TNBS, (B) DIC, (C) NEM, (D) PMSF, and (E) 2-ME on 5′-PDE activity.

As shown in Fig 4A, TNBS was used to modify lysines present in 5′-PDE, resulting in slight decreases in enzyme activity. Upon increases of TNBS concentration to 2 mmol/L, 5′-PDE activity remained at 94%. These results showed that TNBS had little effect on 5′-PDE activity and suggested that lysine was not an essential residue related to 5′-PDE activity. Similarly, other chemicals, such as DIC, PMSF, and 2-ME, were also applied to modify arginines, cysteines, hydroxyl groups, and disulfide bonds in 5′-PDE, respectively (Fig 4). Our findings revealed that none of these residues, groups, or bonds were necessary for maintaining 5′-PDE activity.

### 5′-PDE catalytic characteristics

**Fig 5:**
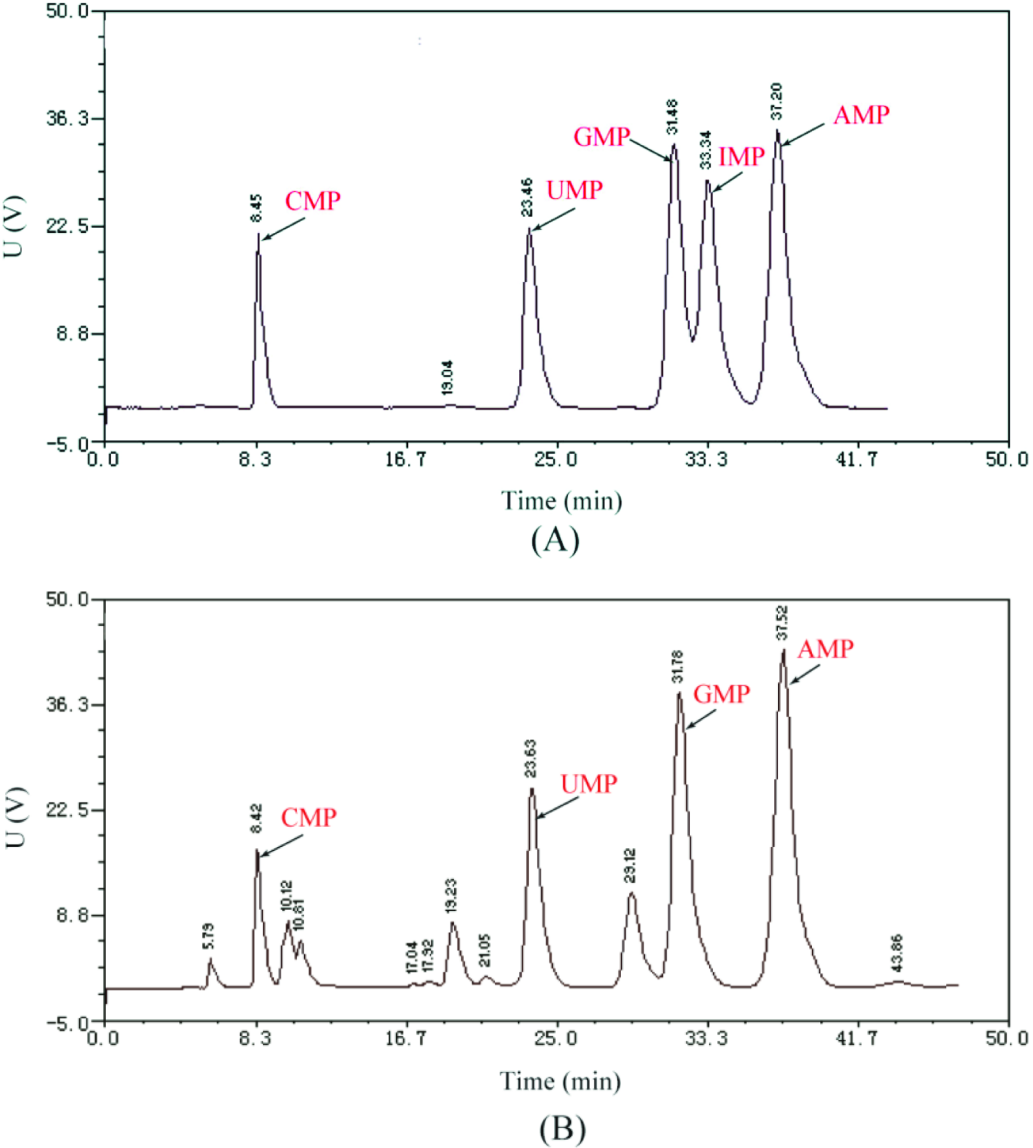
HPLC investigating of 5′-PDE catalytic characteristics. (A) Standard reaction products (CMP, UMP, GMP, IMP, or AMP). (B) RNA hydrolysis by 5′-PDE.

To evaluate 5′-PDE catalytic characteristic, hydrolysis experiments were performed by HPLC using RNA as the substrate. As shown in Fig 5, four types of mononucleotide (CMP, UMP, GMP, and AMP) were the main products of 5′-PDE hydrolysis, with no IMP detected, indicating specific RNA hydrolysis by 5′-PDE.

## Discussion

In this study, we described the isolation of purification of 5′-PDE with excellent catalytic specificity from *A*. *fumigatus*, followed by analysis of important structural characteristics necessary for RNA hydrolysis. Following purification, 5′-PDE exhibited a specific activity of 1036.76 U/mg protein and a molecular weight of 9.5 kDa, as well as optimal temperature and pH of 60°C and 5.0, respectively. However, 5′-PDE activity was partially inhibited by the presence of Fe^3+^, Cu^2+^, Fe^2+^, and Zn^2+^, but slightly improved by K^+^ and Na^+^, which agreed with a previous report [18]. Based on the results of structural modification experiments using different chemicals, it revealed that carboxyl and imidazole groups were essential for enzyme activity based on their locations in the 5′-PDE active site, thereby providing a theoretical basis for potentially improving 5′-PDE enzymatic characteristics by protein engineering. More importantly, 5′-PDE exhibited *V*_max_ and *K*_M_ values of 0.71 mmol/mg·min and 13.60 mg/mL, respectively, which were superior to those of nuclease P1 from *P*. *citrinum* (*K*_M_ was 35.44 mg/mL) [18] and indicated excellent capabilities for RNA hydrolysis. However, there were also two factors to limit the industrial application of 5′-PDE from *A*. *fumigatus*, including: (1) the low efficiency of free-cell fermentation for 5'-PDE production; (2) the security problems for 5′-PDE from *A*. *fumigatus*[21].

Therefore, to improve fermentation performance, immobilized fermentation were proposed and applied to produce 5′-PDE [22]. Compared to free-cell fermentation, immobilized fermentation can prolong enzyme production, obtain higher productivity, and increase operational stability [23]. When immobilized on chitosan nanoparticles and cellulose, the temperature, pH, stability, and utilization of *P*. *citrinum* were effectively improved, which subsequently reduced the cost of nuclease P1 production [24]. However, complex process control, poor performance upon repeated use of the carrier, and complicated downstream processing limit the utility of immobilization for 5′-PDE production. Therefore, most research has focused on developing a more natural and efficient immobilized carrier for 5′-PDE production.

Currently, 5′-PDE has potential commercial applications in the food industry, specifically in the processing of nucleotide-rich food ingredients to enhance flavors in food products, such as umami. Therefore, security problem played an important role in industrial applications for 5′-PDE [21]. Nuclease P1 from *P*. *citrinum*, an anamorphic mesophilic fungus with a history of safe use as a fermentation organism in Europe and Asia, is used in the production of ribonucleases [25]; however, the safety of 5′-PDE from *A*. *fumigatus*, a pathogenic bacteria, for use in food processing should be comprehensively evaluated by means of standard toxicological testing methods, including *in vitro* Ames tests and chromosome-aberration assays and *in vivo* rat erythrocyte micronucleus assays, in future studies.

## Supporting information

S1 Fig The phylogenetic experiments for identifying strain *A*. *fumigatus* XD-9. (a) the amplification of 18S rDNA; (b) the phylogenetic analysis of strain XD-9.
(DOCX)

S2 Fig **Effects of glucose (a) and water content (b) on the enzyme activity of 5'-PDE**.
(DOCX)

S3 Fig SDS-PAGE electrophoresis of 5'- PDE. (DOCX)
