# Supplementary material for: Isolation, purification and characterization of 5'-phosphodiesterase from Aspergillus fumigatus
Source: PLoS One. 2017 Oct 26;12(10):e0186011. doi: 10.1371/journal.pone.0186011 (PMC5657630; doi:10.1371/journal.pone.0186011)
Supplement: S1 Fig — (a) the amplification of 18S rDNA; (b) the phylogenetic analysis of strain XD-9. (DOCX) [file pone.0186011.s001.docx]

**
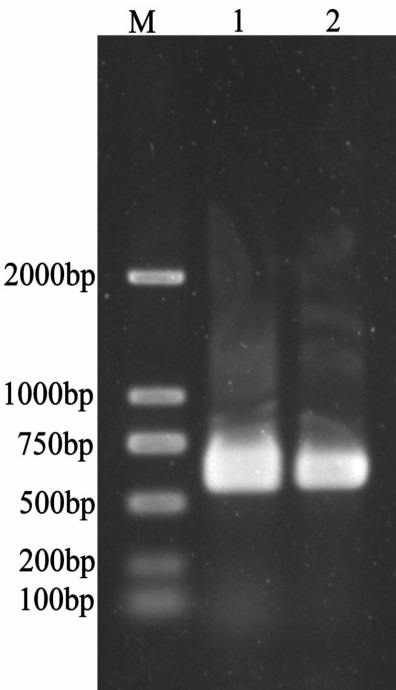
**

S1Fig. The phylogenetic experiments for identifying strain *A. fumigatus* XD-9: (a) the amplification of 18S rDNA; (b) the phylogenetic analysis of strain XD-9.
