# Supplementary figures and images for: Isolation, purification and characterization of 5'-phosphodiesterase from Aspergillus fumigatus
Source: PLoS One. 2017 Oct 26;12(10):e0186011. doi: 10.1371/journal.pone.0186011 (PMC5657630; doi:10.1371/journal.pone.0186011)

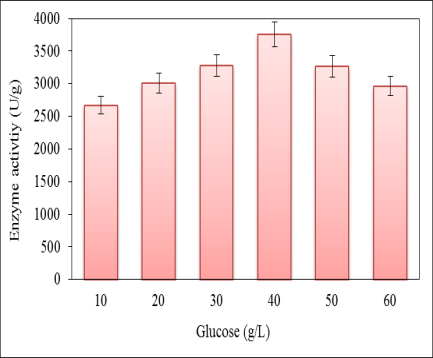

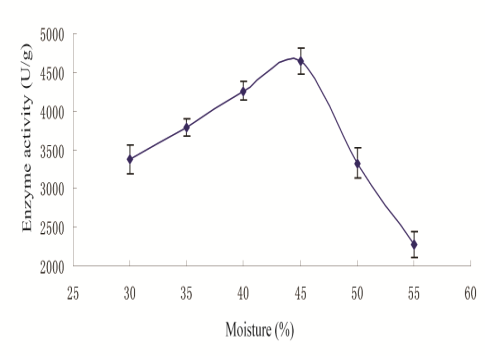


Water content (%)

1. (b)

S2 Fig. Effects of glucose (a) and water content (b) on the enzyme activity of 5'-PDE.

Supplement: S2 Fig — Effects of glucose (a) and water content (b) on the enzyme activity of 5'-PDE. (DOCX) [file pone.0186011.s002.docx]

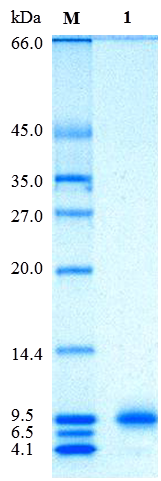


S3 Fig. SDS-PAGE electrophoresis of 5'- PDE

Supplement: S3 Fig — (DOCX) [file pone.0186011.s003.docx]
